# A deep learning-based approach for axle counter in free-flow tolling systems

**DOI:** 10.1038/s41598-024-53749-y

**Published:** 2024-02-10

**Authors:** Bruno José Souza, Guinther Kovalski da Costa, Anderson Luis Szejka, Roberto Zanetti Freire, Gabriel Villarrubia Gonzalez

**Affiliations:** 1 Industrial and Systems Engineering Graduate Program, Pontifical Catholic University of Parana-PUCPR, Curitiba, Paraná 80215-901 Brazil; 2 Pumatronix Electronic Equipment Ltd., Bartolomeu L. de Gusmão 1970, Curitiba, 81650-050 Brazil; 3 Production and Systems Engineering - PPGEPS, Universidade Tecnológica Federal do Paraná-UTFPR, Curitiba, Paraná 80230-901 Brazil; 4 Expert Systems and Applications Lab, Faculty of Science, University of Salamanca, Plaza de los Caídos s/n, 37008 Salamanca, Spain

**Keywords:** Axle counter, Deep learning, Free-flow tolling systems, You Only Look Once, Software, Electrical and electronic engineering

## Abstract

Enhancements in the structural and operational aspects of transportation are important for achieving high-quality mobility. Toll plazas are commonly known as a potential bottleneck stretch, as they tend to interfere with the normality of the flow due to the charging points. Focusing on the automation of toll plazas, this research presents the development of an axle counter to compose a free-flow toll collection system. The axle counter is responsible for the interpretation of images through algorithms based on computer vision to determine the number of axles of vehicles crossing in front of a camera. The You Only Look Once (YOLO) model was employed in the first step to identify vehicle wheels. Considering that several versions of this model are available, to select the best model, YOLOv5, YOLOv6, YOLOv7, and YOLOv8 were compared. The YOLOv5m achieved the best result with precision and recall of 99.40% and 98.20%, respectively. A passage manager was developed thereafter to verify when a vehicle passes in front of the camera and store the corresponding frames. These frames are then used by the image reconstruction module which creates an image of the complete vehicle containing all axles. From the sequence of frames, the proposed method is able to identify when a vehicle was passing through the scene, count the number of axles, and automatically generate the appropriate charge to be applied to the vehicle.

## Introduction

Guaranteeing efficient human mobility in cities brings economic benefits and improves the quality of life, both individually and collectively^1^. The development in transportation considering both structural and operational aspects is important for achieving high-quality mobility. In light of these improvements, the Intelligent Transportation System (ITS) is a concept that has been refined in recent years^2^. The ITS can be represented by a software and hardware system that uses information and communication technology in mobility to improve transportation efficiency from a strategic standpoint. Mobility improves due to its use in terms of security, punctuality, and real-time information^3^.

Advances in image processing considering embedded devices have resulted in significant improvements in image collection, storage, and sharing. This technological development is being exploited in a variety of applications, such as image and video capture where cameras are frequently used to perform analysis, identification, and tracking of objects in real-time^4^. This progress allows for the exploration of solutions in areas that are now getting attention, such as urban mobility and smart cities^5^.

Several examples show how technology applications are relevant components of innovation in urban mobility. There are currently applications in operation, such as traffic light control^6^, smart parking^7^, and traffic flow optimization^8^. Other opportunities for image and video processing are the current toll plaza systems. The possibility of the installation of free-flow tolling would allow transportation managers to perform more efficient operations. In addition to providing benefits from automatic tariff collection, this type of system eliminates the need to stop the vehicle at the toll, reduces both traffic conflicts and traffic jams, and improves the security of the tolling system.

Nowadays, the transport sector has been looking for ways to implement electronic toll collection systems in their most advanced configuration, such as the free-flow electronic toll systems^9^. Free-flow systems are an evolution of electronic systems for automatic vehicle identification, in which gantries installed along a toll road recognize vehicles that cross them and charge them electronically and automatically. The implementation of this system brings productivity gains in toll collection. In addition, it is a viable alternative for the reduction of tariffs since operating costs are reduced. In commercial free-flow tolling systems usually more than one sensor is considered, assuming a multi-sensor approach^10^.

To ensure that the axle counting system works correctly, it is necessary that both the positioning of the objects in the scene and the camera are adequately set up. Given this need, an ideal scenario was defined for the application based on the project requirements of a Brazilian company that develops solutions for mobility on highways and urban environments, where this study was applied.

**Figure 1:**
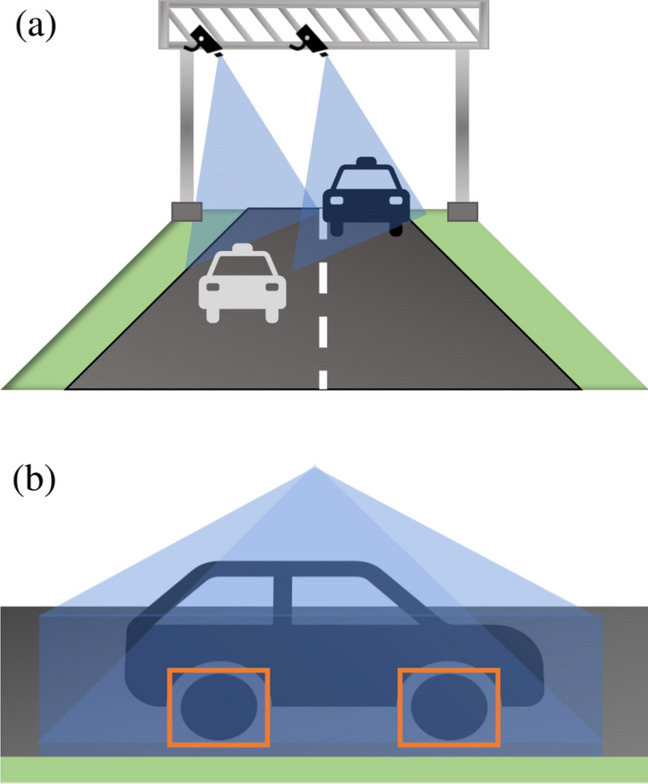
Proposed scenario for axle identification and counting.

In this solution proposal, this scenario is composed of a portico that must cross the road 6 m over the ground. The number of cameras installed on the gantry should be equal to the number of lanes on the highway, as each camera is responsible for analyzing vehicles passing through one of the lanes. The cameras must be positioned on the gantry so that the captured image contains the vehicle axles. A 45-degree positioning is ideal for the highway’s requirements. Figure 1a represents an application scenario on a dual carriageway, while Fig. 1b shows an example of the desired image.

Given this task, this paper proposes an axle counter based on only one sensor, focusing on improving the efficiency of the toll collection system. The proposed approach interprets images through deep learning algorithms to determine the number of axles in the vehicles present in the scene. The main contributions of this paper can be summarized as follows.

- A hybrid method that combines a deep learning structure for classification and a slice image reconstruction process is proposed. The proposed method reduces the necessary number of cameras to be used and automatically generates the charge in a continuous flow toll.
- A dataset, consisting of real and synthetic images, was created and made available in this paper. This dataset was used to train and evaluate the object detector model presented in this research and can be applied in other studies on the subject.
- To achieve the best possible results, the proposed method is based on the most suitable deep learning models, to ensure this outcome, several versions of You Only Look Once (YOLO) are evaluated.

The remainder of this paper is organized as follows: “Related works” describes previous work on computer vision, object detection, and new tolling systems. In “Proposed method”, the proposed method is detailed and the considered dataset is explained. The results of the computations are discussed in “Results and discussions”. Conclusions and also some suggestions for future work are given in “Conclusions and future work”.

## Related works

Automatic toll collection plays an important role in improving highway efficiency and user satisfaction^11^. With the increasing adoption of automatic toll systems worldwide, it is relevant to ensure that vehicle counting is accurate and reliable^12^. Errors in counting can lead to a range of issues, such as incorrect charges, discrepancies in traffic data, and user dissatisfaction^13^. In the case of free-flow tolling, it is important that vehicles and their axles can be correctly detected for the proper functioning of the system.

A promising way to detect objects in images is through the use of computer vision techniques^14^. These techniques can be classical, such as the use of feature detection algorithms like the histogram of oriented gradients^15^, the viola-jones feature descriptor^16^, and texture descriptors^17^, which have been widely used for computer vision tasks and object detection applications.

The use of artificial intelligence-based models is increasing over time^18^, these applications can be focused on classification^19–21^, time series forecasting^22–24^, among others^25–27^. Deep learning approaches are becoming popular^28^, mainly because of their ability to deal with non-linearities^29–31^. As presented by Moreno et al.^32^ the use of hybrid methods can be an outstanding approach in this regard.

Deep learning architectures like Convolutional Neural Networks (CNNs) have promising results for several applications^33^, such as navigation^34^, fault detection^35^, security^36^, image classification^37^, and object detection^38^. The CNNs have proven to be effective in learning high-level representations directly from input data, giving them greater generalization ability and superior performance in object detection tasks^39^. Studies like^40,41^ prove that these applications are promising to be embedded.

Regarding embedded systems, Ref.^42^ presented a power-efficient optimizing framework for field-programmable gate array-based acceleration of the YOLO algorithm. Their framework incorporates efficient memory management techniques, quantization schemes, and parallel processing to enhance performance and reduce energy consumption. In the work of Ge et al.^43^, the YOLOv3 is applied for monitoring full-bridge traffic load distribution. To determine load distribution, the method considers the vehicle’s position, size, and weight, providing a more detailed understanding of the stress distribution on the bridge.

Also using YOLOv3, Rajput et al.^44^ presented an approach for automatic vehicle identification and classification in a toll management system. This approach offers real-time, accurate, and efficient toll collection, thereby enhancing the overall effectiveness and user experience of toll facilities. The system’s adaptability and potential for security enhancements make it a valuable asset for transportation infrastructure.

Deep learning models are becoming increasingly popular in mobility applications. In Ref.^45^, the authors employ YOLOv3 in their vehicle detection and counting system on highways. Similarly, in Ref.^46^, the authors propose an optimized version of YOLOv4 for vehicle detection and classification. Moreover, other applications, such as^47,48^, demonstrate different uses of object detection, including license plate detection, road crack detection, and traffic light recognition respectively.

Zarei et al.^49^ proposed the Fast-YOLO-Rec. This model combines the strengths of the YOLO-based architecture and recurrent-based prediction networks for vehicle detection in sequences of images. Based on YOLO, they localize vehicles in each frame, providing a strong initial detection foundation. In Ref.^50^ the vehicle detection is based on a tiny version of YOLOv5 called T-YOLO. An innovation of T-YOLO is its ability to handle tiny vehicles, which are often challenging to detect due to their limited spatial presence.

To overcome the challenges of limited and imbalanced data, Dewi et al.^51^ employed Generative Adversarial Networks (GANs) to synthesize additional training samples, expanding the dataset and promoting model generalization. In their application, the YOLOv4 and multiple GANs are explored. The resulting model exhibits promising performance across a wide range of traffic sign types, sizes, and orientations, even in challenging environmental conditions.

To perform the axle counting and speed measurement, Miles et al.^52^ applied the YOLOv3. The model was implemented to track the vehicles and assign a wheel to a vehicle if the center point of the wheel bounding box fell within the vehicle bounding box. The outputs from the axle detection were then processed to produce axle counts for each vehicle, achieving an accuracy of 93% across all vehicles where all axles were visible.

Li et al.^53^ also employed a tracking-based method for vehicle axle counting. In their work, the YOLOv5s is utilized for axle detection and classification into double and single-wheeled vehicles. The obtained results indicate that the axle detector achieved a mean Average Precision (mAP) of 95.2%. To address the issue of vehicles sometimes not fitting within a single frame, there are some alternative approaches to the tracking-based method, such as utilizing image stitching. However, performing real-time image stitching can be computationally costly, especially for online applications^54^.

In Refs.^55,56^, the authors focused on the identification of electrical insulators in images taken by unmanned aerial vehicles. They proposed modifications to the base structure of the model to obtain improvements in its performance. In these works, in addition to detecting the chain of electrical insulators, classification is also carried out in case of any visually identifiable defect, such as broken or flash-over insulators.

In Ref.^57^ the application of a modified YOLOv5 model called T-YOLO for vehicle detection was proposed. The images in the dataset used were obtained from a camera with a top view of the parking lot scene, and just as in the case of insulator detection, the vehicles in question end up being relatively small objects in the image, which supports the application of YOLO models for objects which are not necessarily large. In Ref.^58^ the application of a YOLO-v2 for vehicle detection in the Karlsruhe Institute of Technology and Toyota Technological Institute dataset was presented with promising results.

## Proposed method

The proposed method aims to examine a sequence of frames to determine the vehicle’s axle count. The case study scenario consists of a camera installed in a gantry, which will be responsible for analyzing a lane of the highway. In the scenario, the vehicles pass through the camera sequentially at different intervals of time.

The first challenge of the proposed task concerns identifying when a new vehicle enters the scene. To accomplish this task, a passage manager that uses optical flow techniques in conjunction with axle identification was developed to understand when a vehicle is passing through the image. When the system identifies that there is a vehicle on the scene, it analyzes whether a given frame corresponds to a new passage or a passage that is in progress.

**Figure 2:**
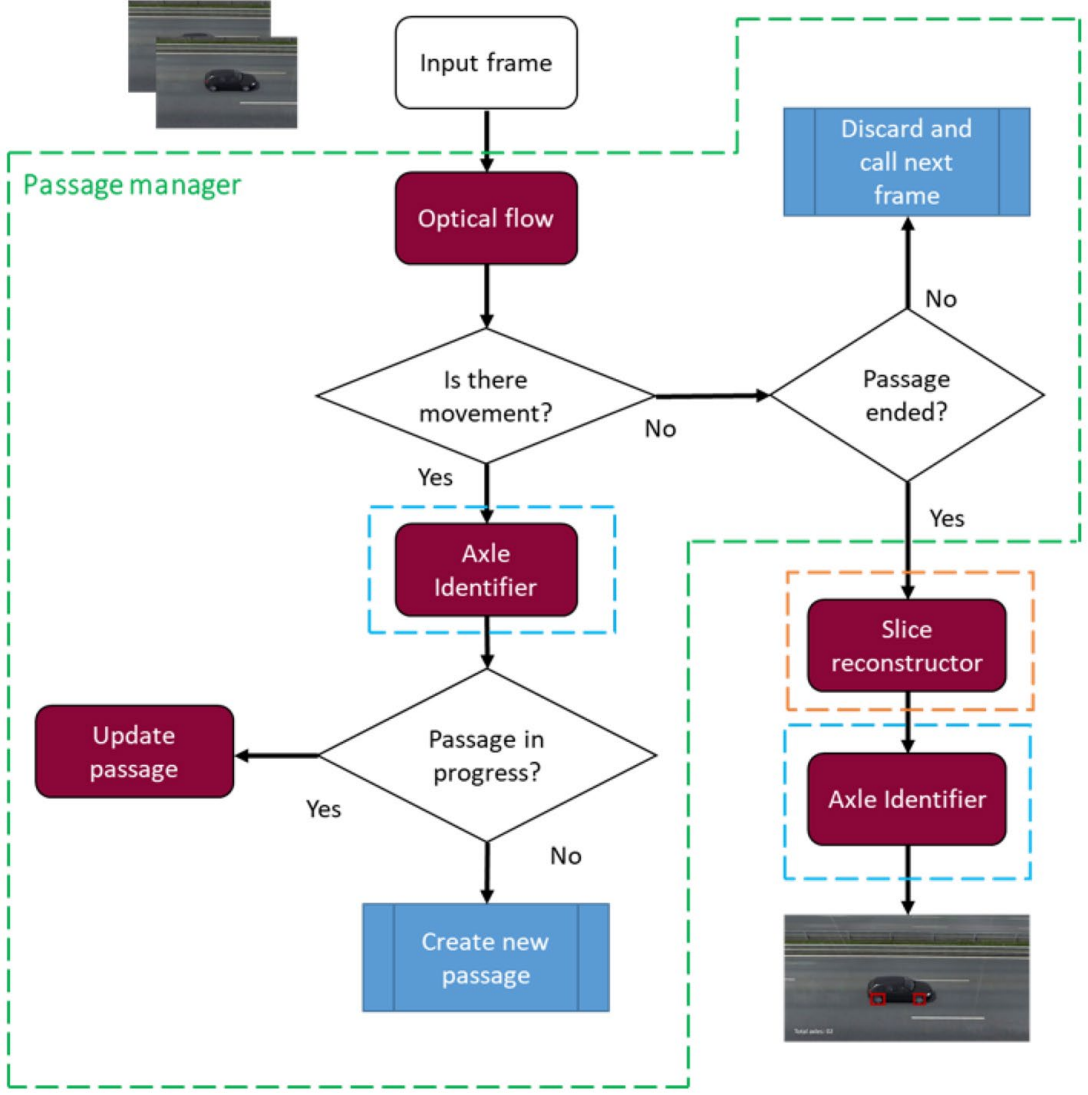
Flowchart of the proposed method.

The system must be consistent in identifying which passage each axis belongs to. The system also needs to be able to save the frames corresponding to a given passage and, at the end of the passage, use these frames to generate a panoramic image, where it is possible to visualize the entire vehicle. The slice reconstruction module performs this process. The last step aims to use the axle identifier in the image generated by the slice reconstructor to perform the vehicle axle count. Figure 2 presents a general flowchart of the pipeline of the proposed approach.

### Axle identifier

To perform the axis identification task, an object detection YOLO model based on deep learning is applied to detect vehicle wheels and axle counting. The YOLO model is a CNN that divides images into grids, with each grid cell detecting objects within its boundaries^55^. This model has a structure that needs only one shot to detect and classify the objects^56^. The model falls under the category of single-stage detectors, as it only requires a single shot through the image to classify and detect the target object^59^.

YOLO models have exhibited promising results for object detection in different areas, for example in Ref.^60^ where the models were used in the health area, or in Refs.^61,62^ where it is used for safety and urban mobility respectively. Since its first version, YOLO has evolved and gained improvements in its architectures, leading to different versions^63^.

The YOLOv5 is integrated with an algorithm known as AutoAnchor. This algorithm assesses and refines anchor boxes that do not suit the dataset and training parameters, such as image dimensions^64^. In September 2022 the YOLOv6 was introduced with a new network structure composed of an efficient backbone employing Re-parameterization Very Deep Convolutional Networks (RepVGG) as its backbone^65^. This novel backbone introduces enhanced parallelism compared to earlier YOLO backbones. Another key improvement was the loss for classification and a loss based on Scylla Intersection over Union (SIoU) or Generalized Intersection over Union (GIoU) for regression^66^.

In the YOLOv7 an extended efficient layer aggregation network was proposed. This enables the models to learn more efficiently by managing the shortest longest gradient path^67^. The YOLOv8 proposes a new solution for diverse visual tasks, spanning object detection, segmentation, pose estimation, tracking, and classification^68^. Drawing inspiration from its predecessor YOLOv5, the YOLOv8 retains foundational elements while imbuing the CSPLayer with new attributes, now called the C2f module^69^.

For a complete evaluation, in this paper, the YOLOv5, YOLOv6, YOLOv7, and YOLOv8 versions are compared. Since there is a trade-off between computational effort and the desired result the medium variation of each model is considered. Considering that YOLOv7 doesn’t have this variation the YOLOv7x is considered. The pseudocode to perform predictions using YOLO is presented in Algorithm 1.

**Algorithm 1:**
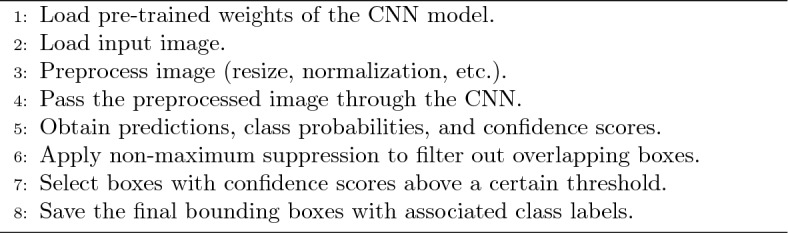
You Only Look Once

### Slice reconstructor

The image slice reconstructor module is responsible for creating an image containing all the vehicle axles present in a given passage. To perform its function, the slice reconstructor takes as input a sequence of frames.

Assuming a scenario in which a vehicle passes through a camera, with horizontal displacement in a single direction, a sequence of frames is obtained where it is possible to get visual information of the complete vehicle. To be able to select the parts necessary to rebuild the vehicle automatically, it was selected the slice from the center of each frame. Since it is considered that the sequence of frames has a complete vehicle passage, which indicates that every part of the vehicle will at some point be located in the center of the image.

Concerning the width of the selected slice, given a sequence of frames, it is possible to consider that the vehicle has a displacement in pixels at each frame. To select the information to rebuild the vehicle without redundancy of information, the ideal scenario is that the width of the slice should be equal to the displacement of the vehicle in pixels.

For the best operation of the algorithm, the theoretical scenario corresponds to a vehicle moving at a constant speed, otherwise, the reconstruction of the slices may show some deformation. However, as this cannot be controlled, the passage manager module performs a speed estimation to select the slice.

**Figure 3:**
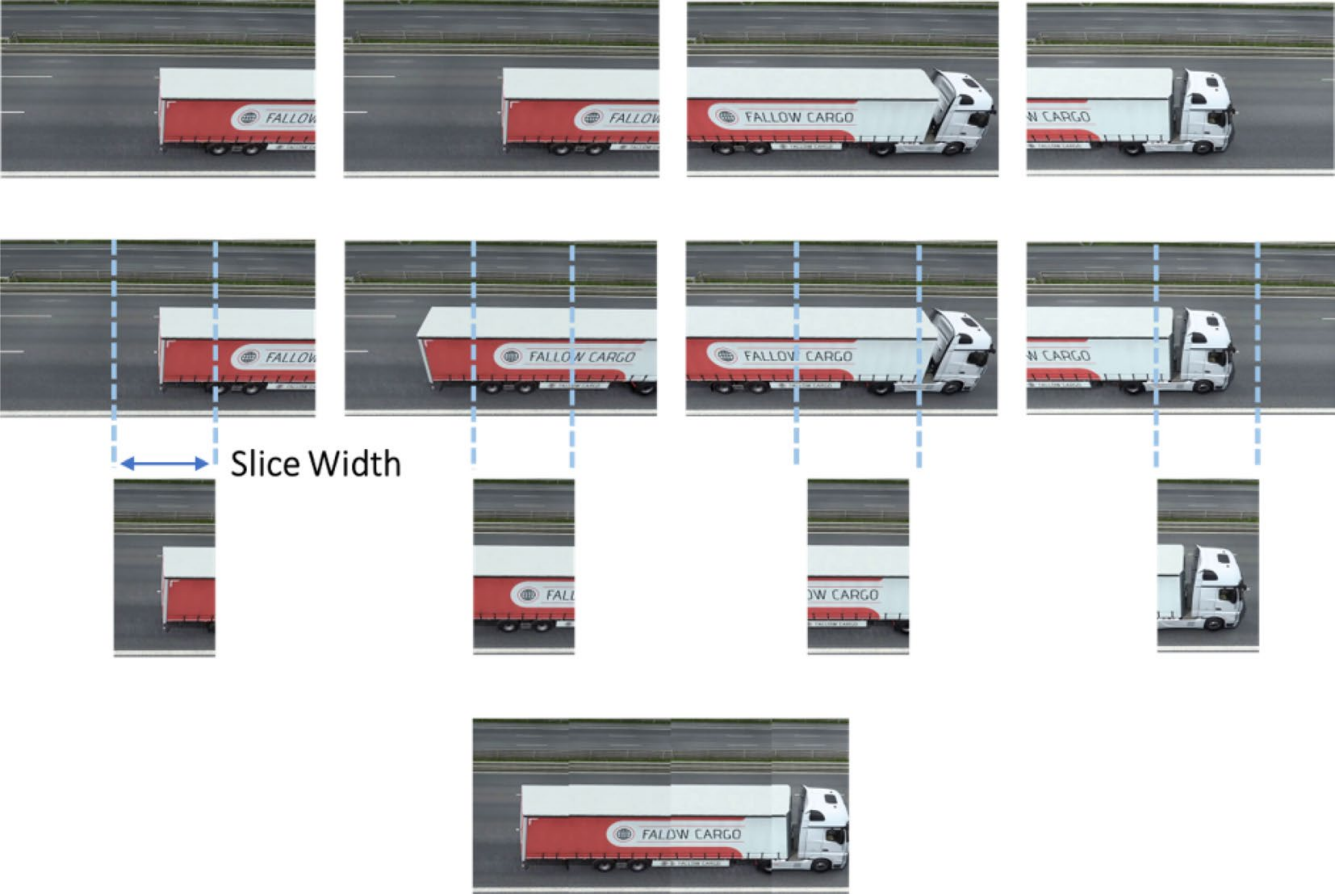
Slice reconstruction process.

During the passage of the vehicle, the average distance of displacement of the object between the frames is calculated. This average distance indicates how many pixels an object moved at each passing frame and corresponds to the slice width. After defining the slice width, the central slice of each frame is concatenated horizontally, creating a complete image of the vehicle. Figure 3 presents how this approach is applied to building the final image.

### Dataset

The dataset created for this project is based on mixed image data, containing real and synthetic images. The dataset was generated and annotated considering its use in different projects, because of this, different classes of vehicles were considered in the annotation stage. Only the wheel category labels will be considered in this research. To have the database for training the object detection model, the company under evaluation installed a camera on a highway to simulate the real scenario of the application. With this, the acquisition of images of vehicles that passed in front of the camera was carried out.

Since detecting axles for large vehicles is part of the scope of this project, the images of bus and truck wheels must be available in the dataset. Due to the lower number of heavy vehicles on the highway, few samples were obtained. To increase the representativeness of the axles of these vehicles in the dataset, the selected alternative was to use synthetic images.

**Figure 4:**
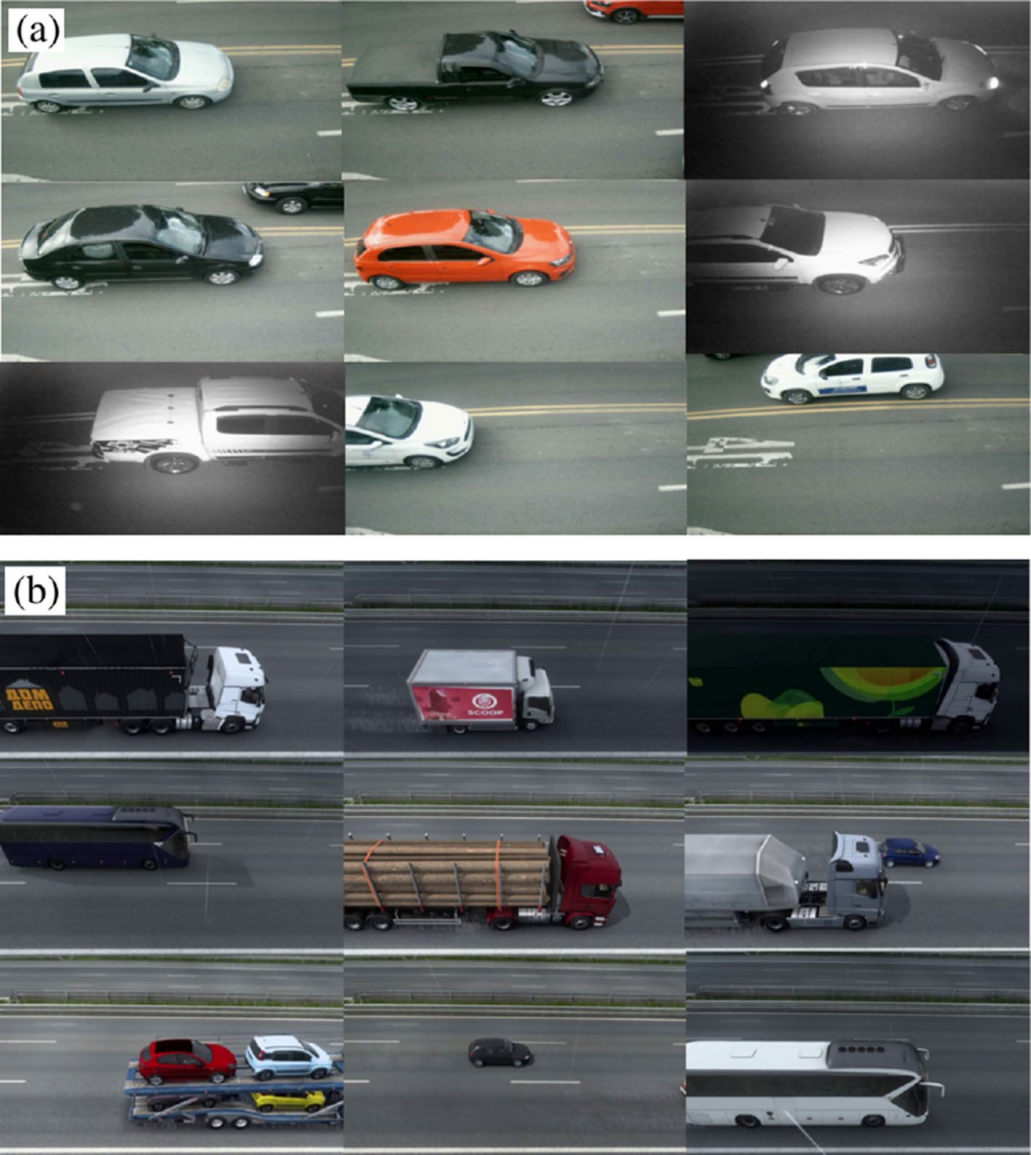
Representation of the mixed dataset: (**a**) real samples, (**b**) synthetic samples.

To create the synthetic data Euro Truck Simulator 2^70^ was used. This simulator was used because it focuses on heavy vehicles such as trucks and buses. Another factor that led to the choice of this simulator was the possibility of camera adjustment. In this simulator is possible to modify the camera’s view, making the captured image at an angle similar to the image of the real application. The use of Euro Truck Simulator 2 was considering the results presented in Ref.^71^, where the authors use this simulator for a vehicle detection application and show that the use of synthetic data can have a positive impact on the generalization of the detection model. Figure 4a,b show real and synthetic samples from the dataset. The division of the dataset is presented in Table 1.

**Table 1:** Data distribution of the dataset.

Images	Training set	Validation set	Test set
Real	968	342	637
Synthetic	3061	685	1163
Total	4029	1027	1800

Focusing on a project feasibility study, it is necessary to analyze the influence caused by the difference between the camera angle of the real installation and the camera angle of the simulator, a fact that also generates a difference in the final size of the vehicles. Given this, only the axles will be considered in the detection task, and the proportion between the vehicles and their respective axles is visually close to the real proportions.

The differences in the proportion of vehicles allow the solution to be analyzed for different camera positions. The dataset is composed of the sum of the real and synthetic data, its distribution is presented in Table 2. All images (original and synthetic) were resized to $640\times 640$ pixels to be standardized for the object detection task. The original images are not publicly available due to being from a private company. The dataset can be requested to the corresponding author on reasonable request. Examples of synthetic data can be found at: https://github.com/Bru-Souza/axles_dataset.

**Table 2:** Distribution of classes in the dataset.

Class	Training objects	Valid. objects	Test objects
Bus	300	99	154
Car	1386	419	876
Motorcycle	61	12	35
Truck	2751	570	1163
Total wheel	9755	2417	4693

### Experiment setup

In the evaluation presented in this paper, an Intel i7-13700K with a graphics processing unit RTX4090 (24GB), and 32GB of random-access memory was considered to perform the experiments. The proposed approach was written in Python language. In this paper, the precision, recall, and mAP metrics are considered.

Precision is defined by the number of positive detections divided by the total number of detections^72^. It is a measure of how often the model predicts correctly, and it indicates how much it’s possible to rely on the model’s positive predictions, given by:

1 $$\begin{aligned} Precison = \frac{TP}{TP+FP}. \end{aligned}$$

where *TP* are the true positives and *FP* are the false positives^73^. The recall is used to evaluate if the model is having missing detections and is given by:

2 $$\begin{aligned} Recall = \frac{TP}{TP+FN}. \end{aligned}$$

where *FN* are the false negatives^74^.

The Intersection over Union (IoU) provides information on the similarity between the region that the algorithm found and the real region of the object present in the image, being defined by the area of the intersection divided by the union of the area of the object and the area detected^75^.

A true positive detection is defined by IoU$>T$, where *T* is a predefined threshold. In this research, the algorithms use $T=0.5$. The mAP is calculated as the weighted mean of precisions at each threshold (given by the IoU), and the weight is the increase in recall from the prior threshold. The equation to calculate the mAP is according to:

3 $$\begin{aligned} mAP = \frac{1}{\Lambda }\sum _{i=1}^{\Lambda } AP _{i} . \end{aligned}$$

where $\Lambda$ is the number of classes.

The mAP incorporates the trade-off between precision and recall. This property makes mAP a suitable metric for most detection applications. Performing the plot of the precision-recall curve is also a way to obtain the mAP. The Average Precision (AP) is defined as the area under the curve, and the mAP is defined as the average of AP of each class^76^. To compare the computational effort of the models the Floating Point Operations (FLOPs) are considered. The considered hyperparameters and main definition setup to compute the experiments are presented in Table 3.

**Table 3:** Hyperparameters and model setup.

Definition	Specification
Language	Python 3
Framework	PyTorch
Main libraries	cv2, numpy, ultralytics, torch
Image size	640$\times$640
Max number epochs	100
Learning rate	0.01
Momentum	0.937
Warmup bias	0.1
Weight decay	0.0005
Optimizer	Stochastic gradient descent

## Results and discussions

In this section, the results will be presented and discussed, the experiments are divided into two parts. The first part aims to evaluate and compare the object detection models, while the second part presents the results regarding axle counting, which is the main focus of this paper.

### Object detection analysis

The performance evaluation of the models is conducted considering a test set of images for this purpose. Table 4 presents a comparison of the results obtained after training the models. To evaluate the compared model, it was considered transfer learning and a fine-tuning process. The transfer learning is based on the pre-trained weights of the COCO dataset^77^.

**Table 4:** Comparative model results.

Model	Precision	Recall	mAP @0.50	mAP @0.50:0.95	FLOPs
**YOLOv5m**	**0.994**	**0.982**	**0.994**	0.800	49.0
YOLOv6m	0.990	0.832	0.989	0.792	82.2
YOLOv7x	0.979	0.923	0.975	0.665	189.9
YOLOv8m	0.992	0.980	**0.994**	**0.820**	78.9

Best performance values are in bold.

All evaluated models were able to achieve values above 0.97 for precision. The best result was obtained by YOLOv5m with a precision of 0.994, while the lowest precision was 0.979 achieved by YOLOv7x. The YOLOv5m achieved also the best performance regarding the recall which was 0.982. The YOLOv6m had the lowest recall in this evaluation.

The YOLOv5m and YOLOv8m models achieved the best mAP result (0.994), and in this measure, the YOLOv7x performed the worst (0.975). Taking into account a wider range of confidence thresholds, ranging from 0.50 to 0.95, the YOLOv8m model exhibited the best performance, reaching a value of 0.820, followed by YOLOv5m with 0.800 and YOLOv6m with 0.792.

The YOLOv5m model showed better performance in most of the metrics. Additionally, it has fewer parameters and fewer FLOPs when compared to the other models considered. Based on these results, the YOLOv5m was selected for axle identification. YOLO has been applied for several tasks by other researchers, some results comparing to our application are presented in Table 5.

**Table 5:** Other researchers’ results.

Author	Application	Model	Precision	Recall	mAP @0.50	mAP @0.50:0.95
Stefenon et al.^55^	Insulator	YOLOv5u	0.981	0.975	0.983	0.905
Souza et al.^56^	Insulator	YOLOv5x	0.983	0.990	0.993	0.957
Han et al.^58^	Vehicle location	O-YOLO-v2	0.940	0.940	0.940	–
Padilla Carrasco et al.^57^	Vehicle location	T-YOLO	0.963	0.996	0.998	0.997
Li et al.^53^	Vehicle wheels	YOLOv3-SPP	0.718	–	0.955	–
Miles et al.^52^	Vehicle wheels	YOLOv3	0.927	–	0.927	–
Our	Vehicle wheels	YOLOv5m	0.994	0.982	0.994	0.800

When comparing the proposed method with other works that also use versions of the YOLO to detect vehicle axes, the camera angle ends up making it difficult to identify all axes since this isometric image ends up causing the occlusion of some axes, compromising the potential of the application. The lateral view of the vehicle is desired because, in addition to facilitating the reconstruction process of the complete vehicle, it generates the possibility of using information about the axle position, such as height and distance between axles, so that in future applications it can differentiate which axles are image belong to the same vehicle, and whether these axles are lowered, raised, or double-wheeled^52^.

### Axle counting analysis

The axle counting analysis concerns the evaluation of the system that receives the sequence of frames and divides these frames into individual vehicle passes, the part that receives the separate frames and generates an image of the complete vehicle, and also the evaluation regarding the number of axles found on each vehicle.

#### Passage manager

The passage manager is the module that, through the optical flow algorithm, can identify and separate vehicle passages in a sequence of frames. The test was performed on real and synthetic images, and for this purpose, a video containing vehicle passages collected in the Euro Truck Simulator 2^70^ simulator was created.

The video that was developed has a total of 30 vehicle passages, of which the system was able to correctly separate 26 passages, obtaining a total of 86.67% accuracy. Concerning the real data, 60 passages of different types of vehicles, such as cars, trucks, and buses were collected, and the system was able to correctly separate 46 passages, obtaining a total of 76.67% accuracy.

#### Reconstruction analysis

**Figure 5:**
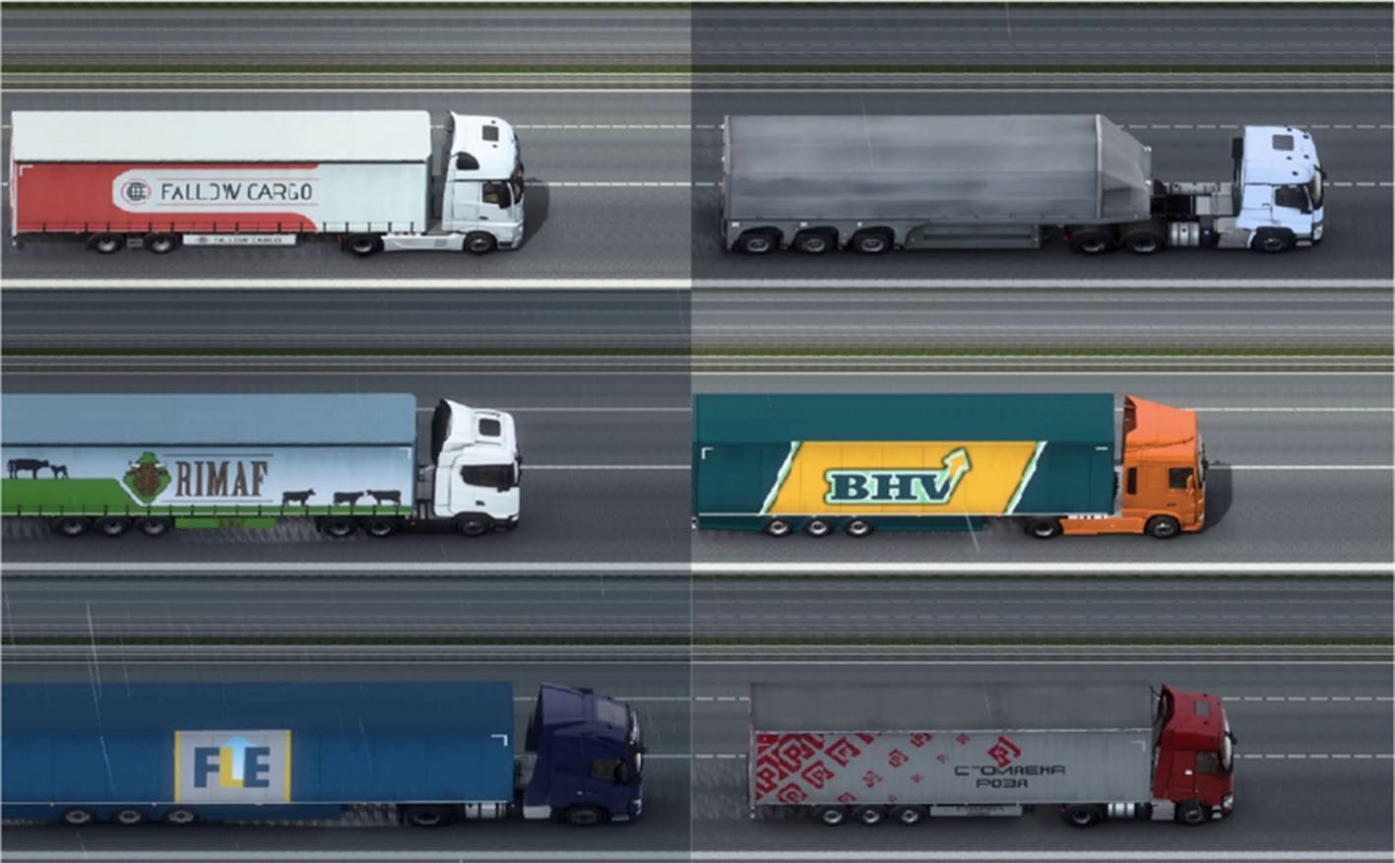
Results from the reconstruction process.

Based on the result of the passage manager module, a set of frames is obtained for a specific vehicle passage. These vehicle passages are then sent to the module responsible for slice reconstruction. Figure 5 presents examples where the algorithm can reconstruct the image without deformations that impair the visual analysis of the vehicle (using images from the Euro Truck Simulator 2^70^).

**Figure 6:**
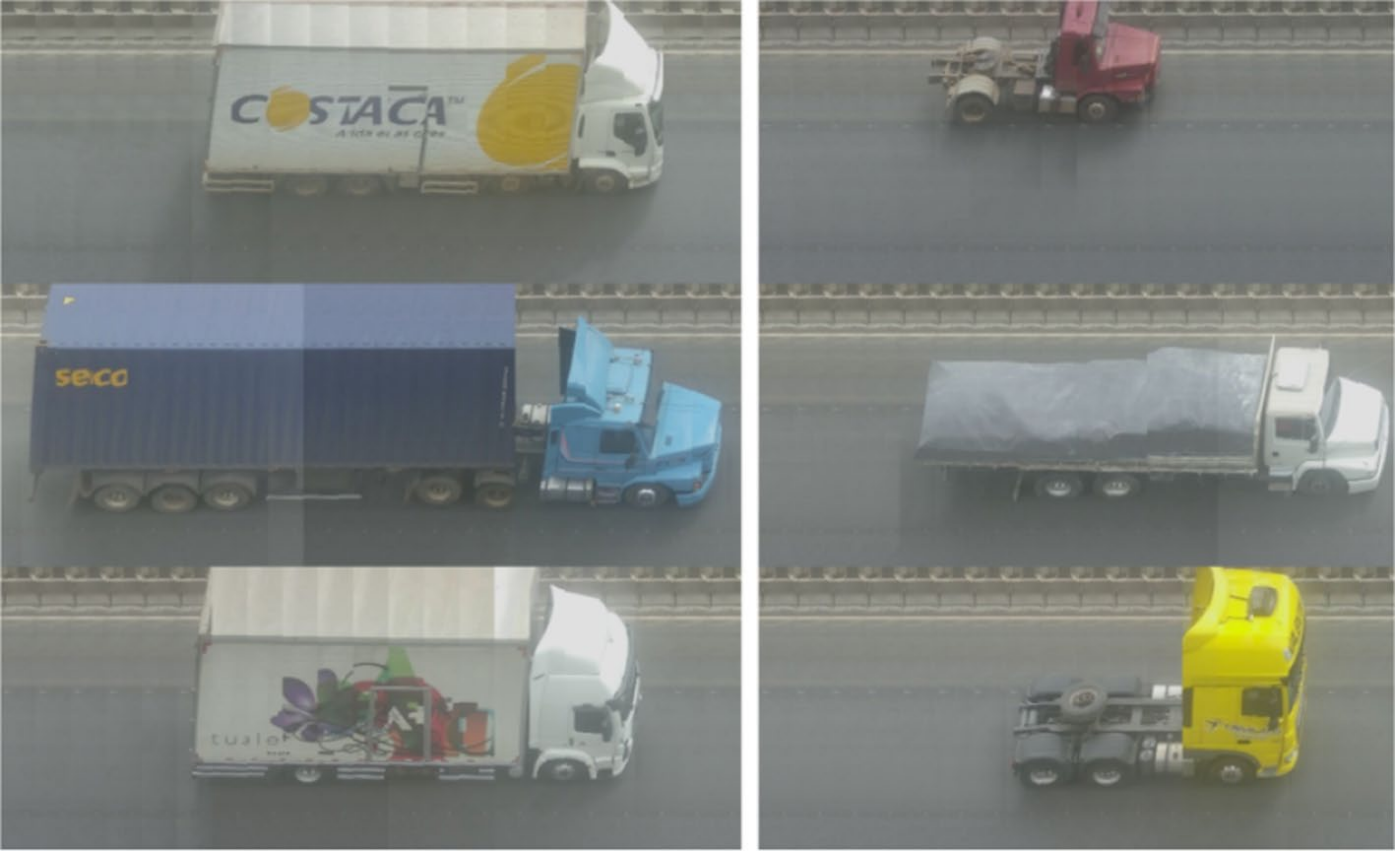
Results from the reconstruction in real images.

Figure 6 presents the results of the slice reconstructor application in a real-world scenario. In this case, for the reconstruction algorithm to function properly, a preprocessing step is necessary on the frames to remove possible camera distortions.

In these examples, the synthetic images had more sharpness than the real images. This happened because it was a cloudy day when the data were recorded, based on this example it is possible to observe that real data have additionally the influence of weather conditions, that in some cases may impair axle identification.

#### Axle counter analysis

**Figure 7:**
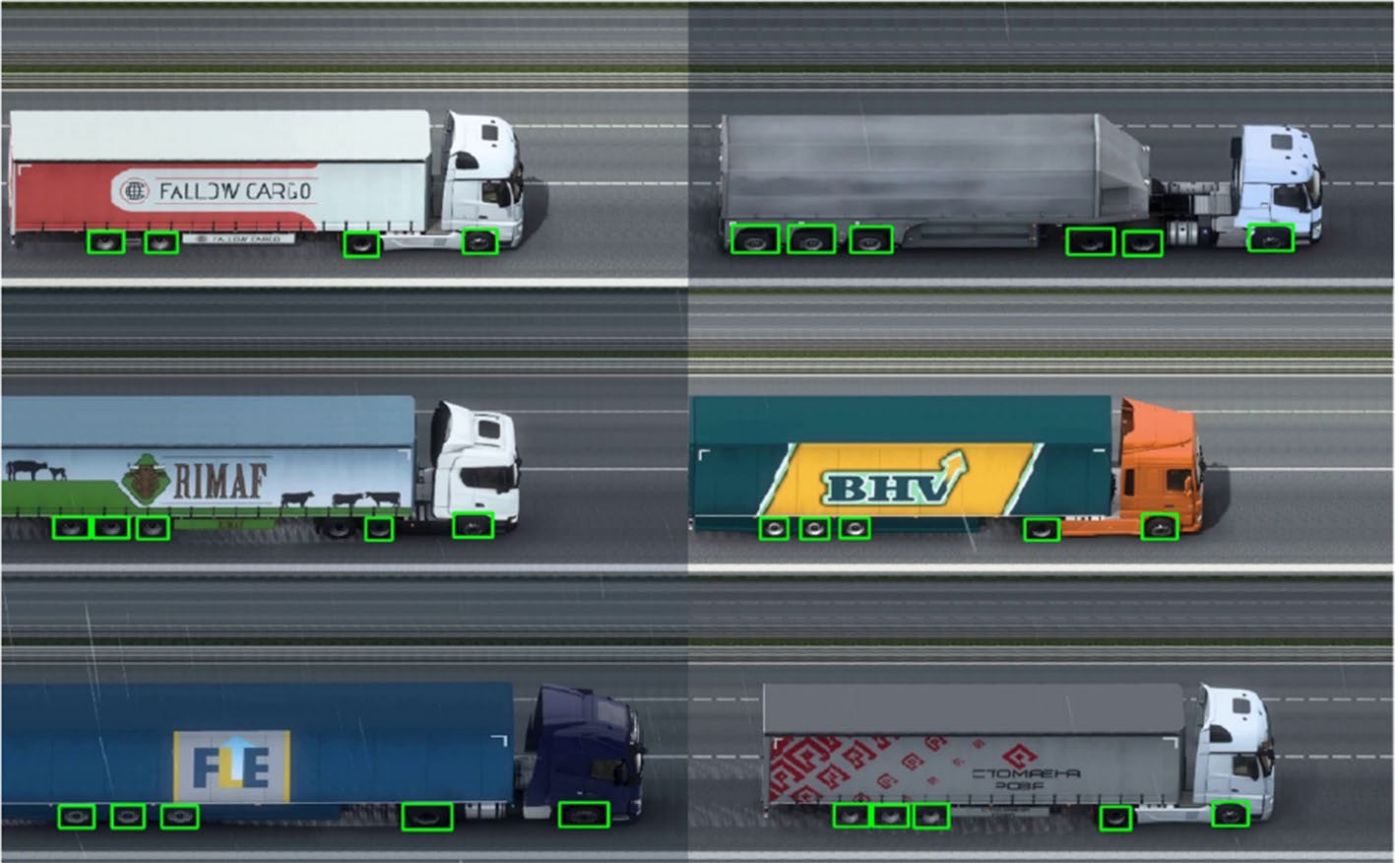
Axle counter inference.

**Figure 8:**
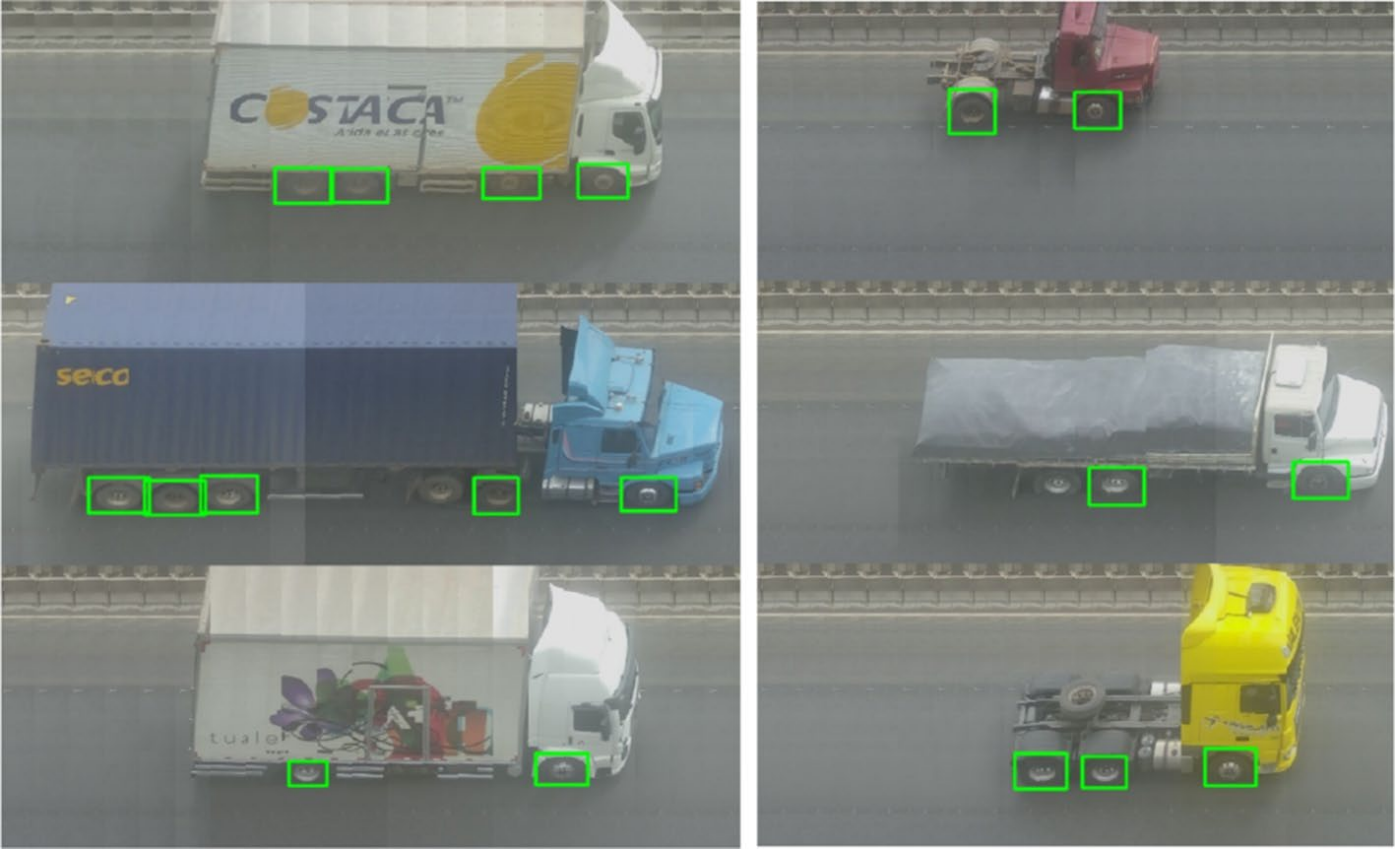
Axle counter inference in real images.

The results of applying the trained model to the reconstructed images can be observed in Figs. 7 and 8, where examples of application on synthetic and real data are shown, respectively. The object detector was able to successfully identify the axles of the vehicles, meeting the needs of this project.

**Limitations**: After the slice reconstruction process, although the height remains the same as the original, the width is defined by the slice width multiplied by the number of frames in the passage. If the image has larger dimensions than the images used to train the model, resizing it for inference can lead to errors in detection results.

## Conclusions and future work

This study has addressed the challenges of implementing an axle counter system based on computer vision. A deep learning model was applied to identify axles, and after that, an algorithm for vehicle management and axle counting was implemented.

The computer vision-based systems have some limitations compared to the system proposed in this paper due to several outliers that may occur in the traffic scenario. The use of deep learning for axle counting proved capable of identifying vehicle axles. The YOLOv5m was selected after comparing this model to the YOLOv6m, YOLOv7x, and YOLOv8m models, where the YOLOv5 family model achieved a precision of 0.994 and a recall of 0.982 during the training process. Furthermore, the values of mAP@0.50 and mAP@0.50:0.95 were 0.994 and 0.800, respectively.

The passage manager module and the slice reconstruction module were developed, enabling the determination of vehicle passages in the scenario and creating an image containing the complete vehicle for wheel counting. Regarding the outcomes of the passage manager obtained during the experiments, the proposed approach had an accuracy rate of 86.67% for synthetic data, correctly identifying 26 out of 30 passage samples, and 76.67% for real-world images, accurately classifying 46 out of 60 passages. These results underscore the effectiveness of the implemented system in reliably managing and processing axle passages, both in synthetic and real-world scenarios.

To enhance the performance of the axle counter, in future works, an alternative would be to include images of the reconstructed vehicle in the training dataset. It would be promising to incorporate images of vehicles in different backgrounds to obtain a more generalizable model. A crucial aspect to improve the robustness of the application is to develop a strategy for handling cases in which one vehicle is carrying another, such as tow and stork trucks.

Future work can also explore other situations, such as traffic congestion, where there is minimal or no displacement of vehicles between two consecutive frames, or scenarios involving a vehicle and a motorcycle passing simultaneously in a single lane. The tariff collection for trucks currently has differences for axles that are lowered, raised, and double-wheeled, factors that can be approached in future works.

## Data Availability

The datasets generated and/or analysed during the current study are not publicly available due to being from a private company but are available from the corresponding author on reasonable request.
